# Strength Matching Method of Face Gear Pair Considering Service Space Limitation to Improve Strength Performance

**DOI:** 10.3390/ma15207081

**Published:** 2022-10-12

**Authors:** Xiaomeng Chu, Hong Zeng, Yanzhong Wang, Yizhan Huang

**Affiliations:** 1 College of Mechanical Engineering and Automation, Liaoning University of Technology, Jinzhou 121001, China; 2 School of Mechanical Engineering and Automation, Beihang University, Beijing 100083, China

**Keywords:** face gear, strength match, electrochemical machining, transmission performance test

## Abstract

To further improve maneuverability and passability, new heavy-duty vehicles place higher demands on the service space and strength performance of transmission systems. The new surface gear transmission stands out for its unique technical advantages, but how to reduce the volume as much as possible under the premise of meeting the strength performance remains difficult to research. In the past, the method of improving the strength performance of the face gear pair has usually been by increasing the parameters and optimizing the tooth profile. These methods are not suitable for use considering space constraints and guaranteeing center-to-center distances. To overcome the contradiction between small volume and large load, this work proposes a strength matching method to improve the face gear pair’s strength performance in limited service space. First, according to the meshing principle of the face gear pair, the displacement coefficient is considered in the configuration process of the face gear pair, and the mathematical model of the face gear pair is established. Second, to ensure the effective contact area of the face gear pair, a mathematical model of the reverse contact trace avoiding the undercutting and pointing area is established. The proposed method is validated by electrolytic machining and transmission performance tests. This research solves application problems, such as the strength mismatch of the face gear transmission system, and lays the foundation for the engineering application of face gear.

## 1. Introduction

Face gear transmission has become more valued by research institutions and application manufacturers because of its unique technical advantages. Under the condition of volume limitation, in order to improve the strength of the pinion, it is necessary to design the profile shift of the face gear pair. Due to space constraints, it is difficult to match the strength of the face gear pair, and a uniform adjustment of the tooth profile and tooth surface is required. After the design is completed, there is still a problem regarding test verification under heavy load conditions.

In view of the unique technical advantages of face gear transmission, scholars have carried out significant research pertaining to face gear tooth surface design, mechanical characteristics analysis of materials, and manufacturing. Litvin et al. considered two versions of face gear drive geometry with a helical pinion, and a face gear drive with a spur pinion is a particular case of the developed theory [1]. With the purpose of reducing the sensitivity of the gear drive to misalignments, Zanzi et al. investigated an enhanced approach for the application of longitudinal plunging in the manufacturing of a double crowned pinion of a face gear drive [2]. Li et al. proposed a face gear tooth model that is not based on conjugation theories, and derived the extreme geometry boundary conditions of face gear teeth [3]. Huang et al. presented a novel relaxation-free analytical method for residual stress prediction in orthogonal machining, based on inclusion theory, which provides a solution with a clear physical mechanism [4]. Lin et al. proposed a calculating method for tooth root bending strength, to solve the issue of root bending strength in the eccentric straight and helical curve face gear [5]. Zschippang et al. developed a general method for the generation of face gears with helix angle, shaft angle, and axle offset [6]. Zhou et al. presented an accurate measurement model of the face gear tooth surface, wherein the digital tooth contact analysis is implemented with a robust algorithm [7], and they further applied analysis and compensated according to the idea of closed-loop machining [8,9]. Ma et al. analyzed the effect of thermal and mechanical loads on the formation process of residual stress, and specified the effect of thermal and mechanical loads [10].

Mo et al. studied the changing laws of load-sharing coefficients, influenced by flexible support when the sun gear is floating and when the sun gear is normally supported [11]. They also studied the multi-power face gear split flow system [12]. To enhance the finished machining surface quality of face gear, Ming et al. proposed a machining parameter optimization method [13]. Dong et al. introduced a concentric face gear torque split system, used in helicopter main transmissions to transfer more power, and reduce the structure weight [14]. Hochrein et al. offered a direct calculation method for the pointwise geometry of the face gear flank [15]. Pathak et al. proved that honing gear hardness was an important feature for improving the surface quality and microgeometry of the gears [16]. By studying the material characteristics of the spiral bevel gear, Wang et al. studied the material characteristics of the face gear, and proposed a method for the analysis of residual stress and roughness with disk wheel grinding face gear and spiral bevel gear [17,18].

Many scholars have studied the strength analysis and verification of the face gear pair, but research on the strength performance of the face gear pair under space constraints is relatively small. The problem mainly comprises two aspects. First, the tooth surface of the face gear is a high-order complex surface in space, and the pointing and undercutting limit the effective tooth width. Second, the tooth morphology of the face gear and the pinion is quite different. On the premise of ensuring the installation center distance is unchanged, the strength performance of the face gear and the pinion must be matched to resist the transmission meshing impact.

This paper proposes the strength matching method of the profile-shifted face gear pair, which solves the face gear application problem under the condition of small volume and narrow space. First, based on the forming principle of face gear tooth surface, the tooth surface mathematical model of the profile-shifted face gear is established. Second, in order to further improve the strength performance of the face gear, a modification control method for the reverse contact trace is proposed. Then, according to the strength matching model, the optimal strength matching parameters of the face gear pair under different parameters, such as displacement coefficient, are obtained. Finally, the face gear’s electrochemical machining and transmission performance tests are conducted, and the test results verify the correctness of the research method.

## 2. Design of the Profile Shifted Face Gear Pair

### 2.1. Rack Cutter Equation

**Figure 1:**
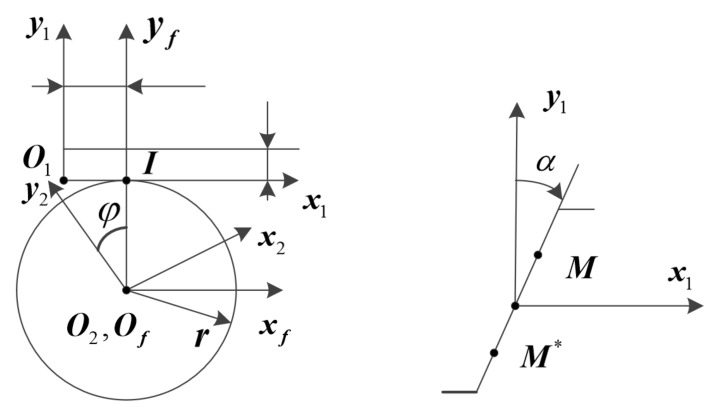
Machining coordinate system of profile-shifted pinion and profile-shifted shaper.

The tooth surface of the profile-shifted pinion and the profile-shifted shaper is derived from the rack tooth surface. The rack tooth profile is a straight line. As shown in Figure 1, the rack tooth profile can be expressed in the coordinate system *S*_1_ by the following formula:

(1) $$\left\{\begin{array}{l} x_{1}=u\sin\alpha \\ y_{1}=u\cos\alpha \end{array}\right.\begin{array}{cc} & -u_{1}<u<u_{2} \end{array}$$

where, *α* is the pressure angle. *u* is the variable parameter, which is used to determine the position of the flow point on the tooth profile (for point *M*, u>0; for point $M^{*}$, u<0). The unit normal vector of the rack tooth surface is:

(2) $$n_{1}=\left(\cos\alpha ,\;-\sin\alpha ,\;0\right)$$

### 2.2. Tooth Surface Equation of Profile Shifted Pinion and Profile Shifted Shaper

Since the derivation process of the tooth surface of the displacement pinion and the displacement gear cutter is the same, the tooth surface equation is derived by taking the displacement gear cutter as an example. *I* is the instantaneous rotation center, *x* is the displacement coefficient, and when *x* = 0, it means no displacement. *m* is the modulus, *φ* is the gear angle, and the displacement s of the rack tool has a relationship with the gear angle *φ*: s=rφ.

The tooth surface equation of the profile shifted shaper can be expressed as:

(3) $$r_{2}=M_{21}r_{1}=M_{2f}M_{f1}r_{1}$$

(4) $$f\left(\begin{array}{cc} u, & \varphi \end{array}\right)=n_{1}\cdot v_{1}^{\left(12\right)}=0$$

where, $r_{1}$ represents the rack tooth surface equation, $r_{2}$ represents the displacement gear tooth surface equation; $M_{21}$ is the coordinate transformation matrix of $S_{1}$ to $S_{2}$, $f\left(\begin{array}{cc} u, & \varphi \end{array}\right)$ is used to represent the meshing equation, $v_{1}^{\left(12\right)}$ is the relative speed.

**Figure 2:**
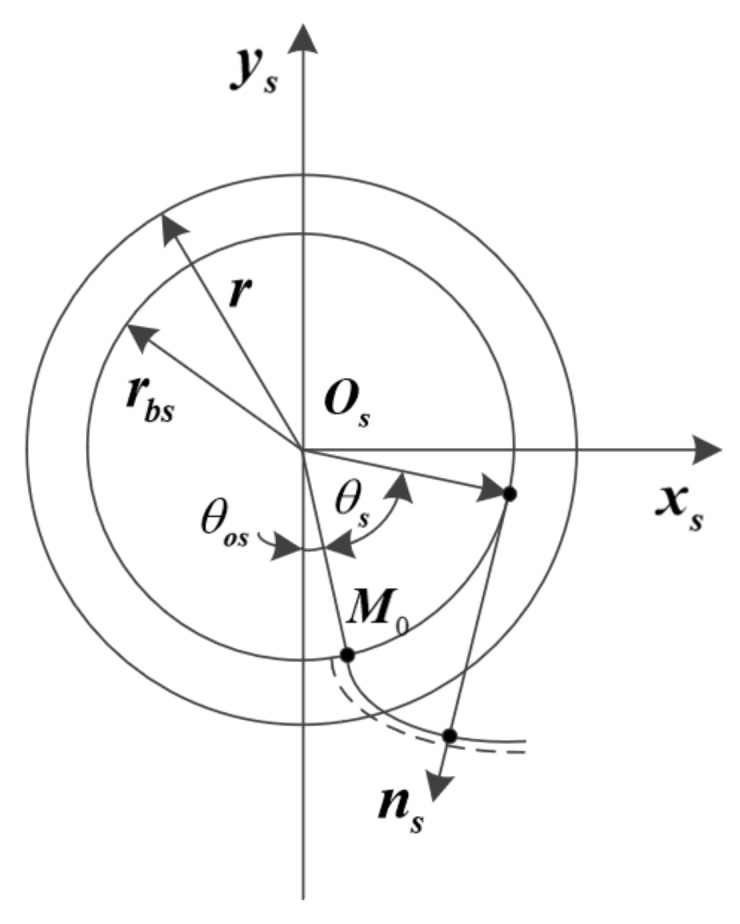
Shaper involute tooth profile.

To derive the tooth surface equation of the face gear, as shown in Figure 2, the tooth profile involute of the profile-shifted shaper is represented in the coordinate system $S_{s}$, and the broken line indicates the involute tooth profile after the displacement. Through coordinate transformation and parameter substitution, the tooth surface equation of the modified gear shaping cutter can be obtained as:

(5) $$r_{s}\left(u_{s},\theta_{s}\right)=\left[\begin{array}{c} r_{bs}\left[\sin\left(\theta_{os}+\theta_{s}\right)-\theta_{s}\cos\left(\theta_{os}+\theta_{s}\right)\right]\\ -r_{bs}\left[\cos\left(\theta_{os}+\theta_{s}\right)+\theta_{s}\cos\left(\theta_{os}+\theta_{s}\right)\right]\\ u_{s}\\ 1 \end{array}\right]$$

(6) $$\theta_{os}=\frac{\pi }{2N_{s}}-\left(\tan\alpha -\alpha \right)-\frac{mx\tan\alpha }{r_{s}}$$

where, $r_{bs}$ is the base circle radius of the shaper, $u_{s}$ is the tooth surface parameter marked in the $z_{s}$ direction, $\theta_{s}$ is the involute parameter, $\theta_{os}$ is the parameter used to determine the width of the tooth groove of the shaper on the base circle, $r_{s}$ is the reference radius of the shaper, $N_{s}$ is the number of shaper teeth.

### 2.3. Tooth Surface Equation of Profile Shifted Face Gear

**Figure 3:**
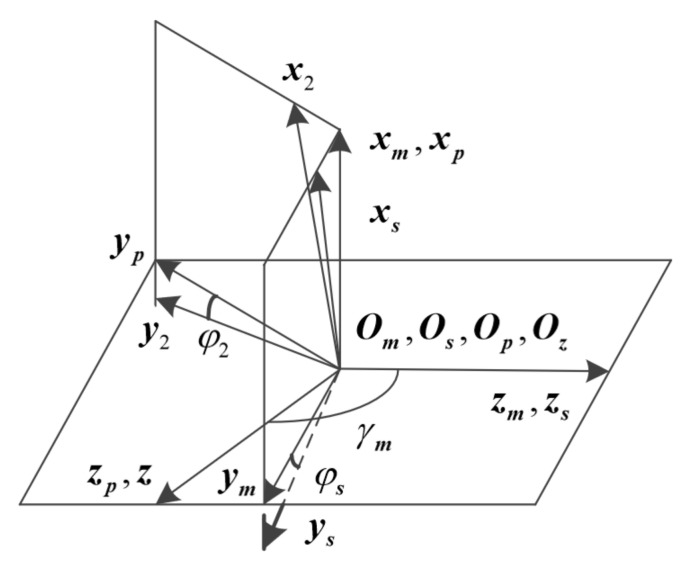
The coordinate systems of shaper machining face gear.

The tooth surface of the face gear is formed by the tooth envelope of the shaper. Using the coordinate system shown in Figure 3, the face gear tooth surface is derived. The rotating coordinate system $S_{s}$ and $S_{2}$ are rigidly connected to the shaper and the face gear, and the auxiliary coordinate systems $S_{m}$ and $S_{p}$ are rigidly connected to the frame. The angle $\gamma_{m}=180^{{}^{\circ}}-\gamma$ is the crossed axis angle of the face gear drive.

In coordinate system $S_{2}$, the tooth surface equation of the profile shifted face gear is expressed as:

(7) $$\begin{array}{l} r_{2}\left(u_{s},\theta_{s},\varphi_{s}\right)=M_{2s}\left(\varphi_{s}\right)r_{s}\left(u_{s},\theta_{s}\right)\\ f\left(u_{s},\theta_{s},\varphi_{s}\right)=n^{\left(s\right)}\cdot v^{\left(s2\right)}=0 \end{array}$$

where, $f\left(u_{s},\theta_{s},\varphi_{s}\right)$ is used to represent the meshing equation, $v^{\left(s2\right)}$ is the relative speed, $M_{2s}$ is the conversion matrix.

## 3. Profile Shifted Face Gear Modification and Strength Analysis

### 3.1. Bidirectional Modification Design of Profile Shifted Face Gear

To improve the load capacity and transmission performance of face gear, a reverse design method of face gear contact trace is proposed to avoid the cutting region of the tooth base.

**Figure 4:**
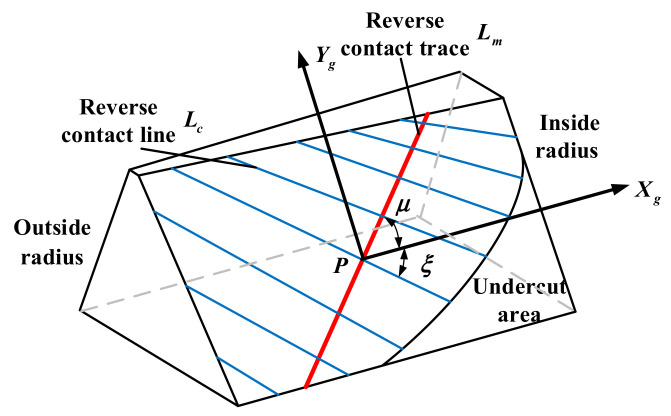
Schematic diagram of face gear tooth surface in coordinate system Sg.

The tooth surface is projected into a fixed coordinate system, and $S_{g}\left(X_{g},Y_{g}\right)$ is the coordinate system of the face gear tooth surface after projection, as shown in Figure 4. *P* is the coordinate origin, and the movement of the contact trace can be realized by controlling the change of its position. The equation of the tooth surface point in the coordinate system $S_{g}\left(X_{g},Y_{g}\right)$ is:

(8) $$\left\{\begin{array}{l} x_{g}=\sqrt{x_{2}^{2}+y_{2}^{2}}-\sqrt{x_{2p}^{2}+y_{2p}^{2}}\\ y_{g}=z_{2}-z_{2p} \end{array}\right.$$

where, coordinates $x_{2}$, $y_{2}$, and $z_{2}$ represent the position of the tooth surface point in coordinate system *S*_2_; coordinates $x_{2p}$, $y_{2p}$, and $z_{2p}$ represent the position of the *P* point on the tooth surface in coordinate system *S*_2_.

The midpoint of the tooth surface of the face gear is selected as *P* point, and coordinate axes *t_g_* and *l_g_* are established along the direction of contact trace *L_m_* and its vertical direction, as shown in Figure 4. According to the relationship shown in Figure 4, it can be calculated as follows:

(9) $$t_{g}=x_{g}\cos\mu +y_{g}\sin\mu$$

(10) $$l_{g}=-x_{g}\sin\mu +y_{g}\cos\mu$$

where, *μ* represents the angle between the reverse contact trace *L_m_* and the projected coordinate axis *X_g_*, and *ξ* represents the angle between the tooth contact line *L_c_* and the projected coordinate axis *X_g_*, which can be calculated by coupling Equations (9) and (10). Furthermore, the pre-control of the tooth face reverse contact trace can be achieved by changing the position of point *P* on the reverse contact trace and the magnitude of the angle parameter *μ*.

**Figure 5:**
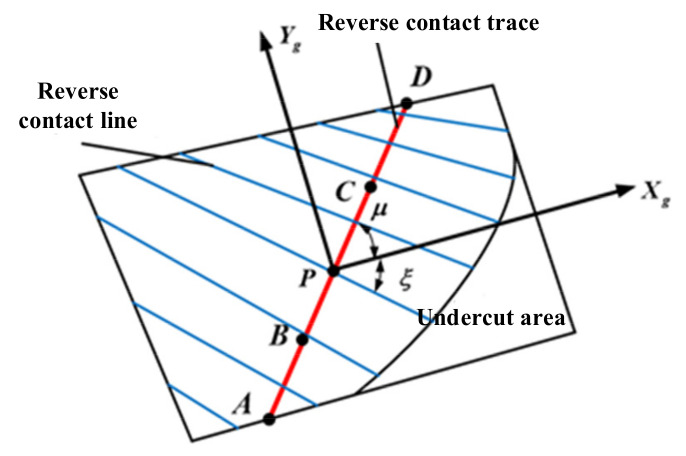
Face gear tooth surface projection.

In Figure 5, there are five pre-positioning points for modification. *A* is the intersection of the preset reverse contact trace and the tooth bottom boundary, *D* is the intersection of the preset reverse contact trace and the tooth top boundary, *B* is the midpoint of points *A* and *P*, and *C* is the midpoint of points *D* and *P*. Furthermore, the preset reverse contact trace curve can be expressed as a fourth-order polynomial function.

The amount of modification in the contact direction is:

(11) $$\delta_{v}=a_{v0}+a_{v1}t_{g}+a_{v2}t_{g}^{2}+a_{v3}t_{g}^{3}+a_{v4}t_{g}^{4}$$

where, (*i* = 0~4) is the undetermined modification coefficient.

**Figure 6:**
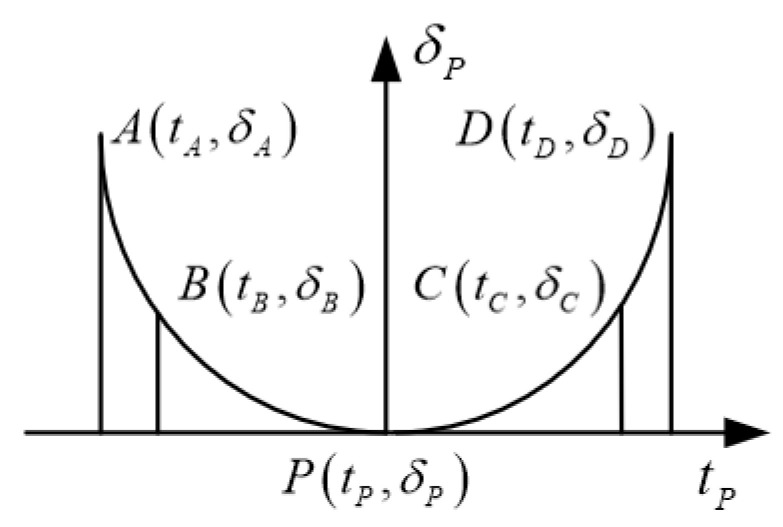
Modification amount of each point in the direction of tooth surface contact trace.

Further, the modification amount of the four points *A*, *B*, *C,* and *D* needs to be designed according to the specific design and transmission performance requirements. The modification coefficient is completed under the premise of determining the modification amount of each point, which is shown in Figure 6. The calculation formula is as follows:

(12) $$\left[a_{v}\right]={\left[M_{v}\right]}^{-1}\left[\delta_{v}\right]$$

where, ${\left[a_{v}\right]}^{T}=\left[\begin{array}{ccccc} a_{v0} & a_{v1} & a_{v2} & a_{v3} & a_{v4} \end{array}\right]$, ${\left[\delta_{v}\right]}^{T}=\left[\begin{array}{ccccc} \delta_{A} & \delta_{B} & \delta_{P} & \delta_{C} & \delta_{D} \end{array}\right]$, ${\left[M_{v}\right]}^{T}=\left[\begin{array}{ccccc} 1 & t_{A} & t_{A}^{2} & t_{A}^{3} & t_{A}^{4}\\ 1 & t_{B} & t_{B}^{2} & t_{B}^{3} & t_{B}^{4}\\ 1 & t_{P} & t_{P}^{2} & t_{P}^{3} & t_{P}^{4}\\ 1 & t_{C} & t_{C}^{2} & t_{C}^{3} & t_{C}^{4}\\ 1 & t_{D} & t_{D}^{2} & t_{D}^{3} & t_{D}^{4} \end{array}\right]$.

To further reduce the sensitivity of the transmission error of the face gear and improve the transmission efficiency, it is also necessary to carry out a modification design along the direction of the tooth surface contact line. The calculation formula is as follows:

(13) $${l^{\prime }}_{g}=\frac{l_{g}}{\sin\left(\mu +\xi \right)}$$

where, ${l^{\prime }}_{g}$ is the amount of variation. ${l^{\prime }}_{g}$ represents the distance from the semi-major axis of the contact ellipse to *P* in the direction of the contact line.

**Figure 7:**
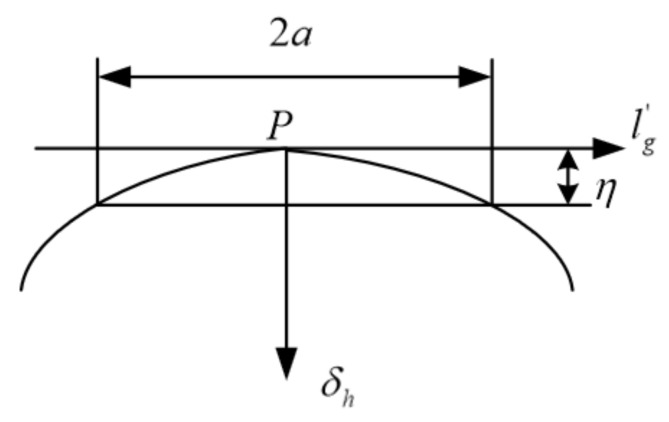
Modification curve in the direction of the contact line.

The amount of modification in the direction of the contact line is shown in Figure 7. The calculation formula is as follows:

(14) $$\delta_{h}=\frac{\eta }{a^{2}}l_{g}^{\prime 2}$$

where, according to the requirements of the meshing performance of the face gear transmission, the size of the face gear tooth surface contacting the ellipse major semi-axis a is preset. *δ_h_* is the amount of modification along the contact line of the tooth surface. The elastic deformation of the tooth surface of the face gear is taken as *η* = 0.00635 mm.

The sum of the modification of the tooth surface profile includes: the modification along the reverse contact trace of the tooth surface, and the modification along the contact line of the tooth surface. The total amount of face gear modification is:

(15) $$\delta =\delta_{v}+\delta_{h}$$

Therefore, the tooth surface equation after modification of the reverse contact trace and contact line is:

(16) $$r_{2}^{d}\left(\phi_{s},\theta_{ks}\right)=r_{2}^{}\left(\phi_{s},\theta_{ks}\right)+n_{2}^{}\left(\phi_{s},\theta_{ks}\right)\cdot \delta$$

### 3.2. Strength Analysis of Face Gear Pair before and after Shifting

**Figure 8:**
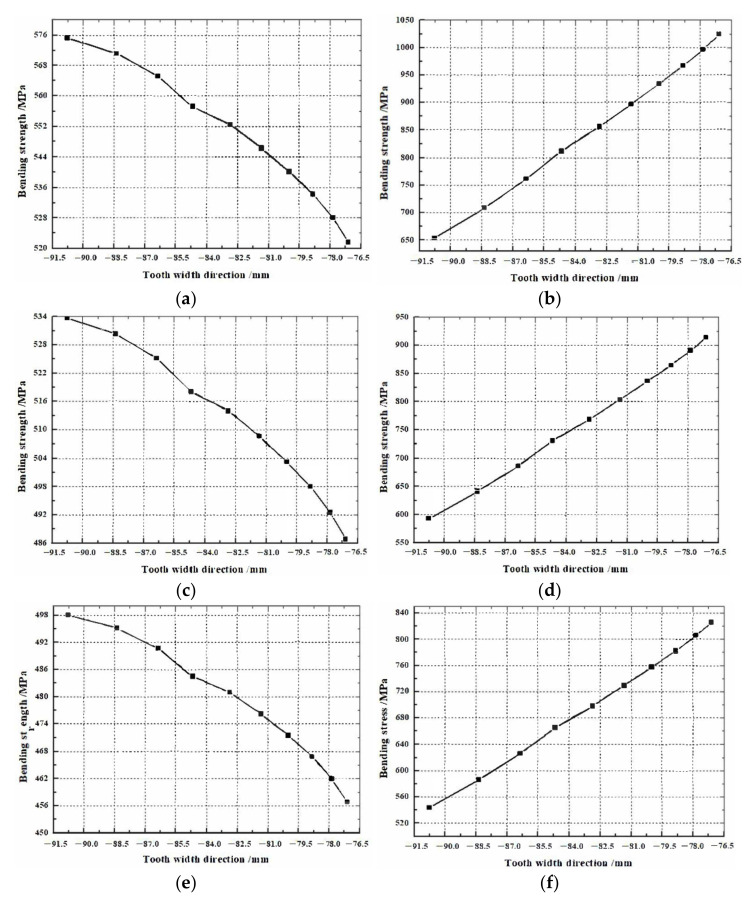

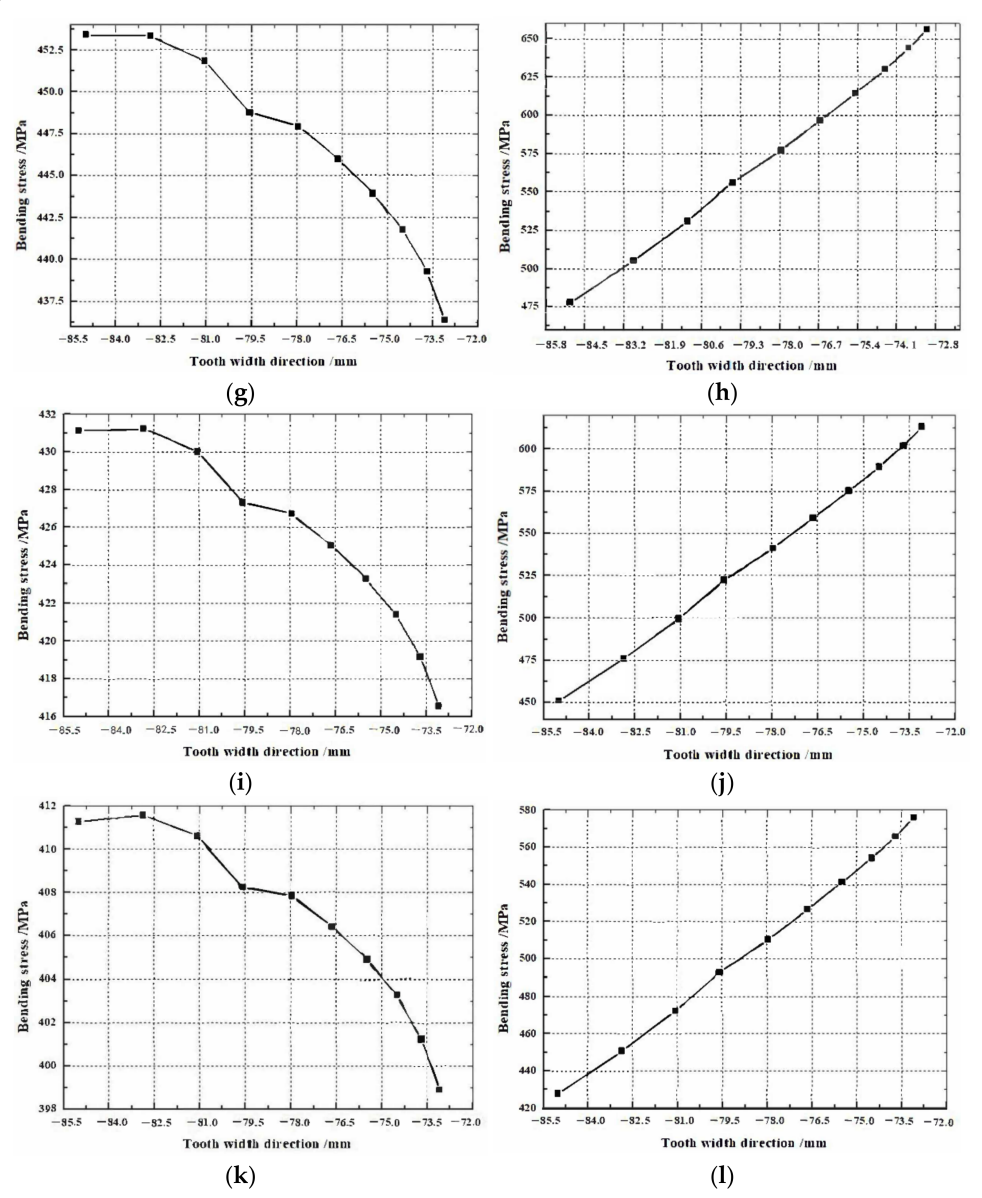
Bending strength of face gear and pinion under different cases: (**a**) face gear bending stress of case 1; (**b**) pinion bending stress of case 1; (**c**) face gear bending stress of case 2; (**d**) pinion bending stress of case 2; (**e**) face gear bending stress of case 3; (**f**) pinion bending stress of case 3; (**g**) face gear bending stress of case 4; (**h**) pinion bending stress of case 4; (**i**) face gear bending stress of case 5; (**j**) pinion bending stress of case 5; (**k**) face gear bending stress of case 6; (**l**) pinion bending stress of case 6.

Based on the above research, the strength performance of the face gear after the displacement is analyzed. The influence of different modification coefficients and gear parameters on the strength performance of face gear pairs is studied. Without changing the volume space occupied by the face gear pair, the design parameters of the face gear pair are shown in Table 1. Using scientific computing software to program the strength comparison analysis of the design parameters of the face gear pair, the strength of the face gear pair under different modification parameters and other conditions is shown in Figure 8. The displacement design adopts the positive displacement of the pinion and the negative displacement of the face gear.

The preset face gear modification parameters are: contact ellipse major axis is 2a=12 mm; contact trace inclination angle is $\mu =75^{{}^{\circ}}$; *A* point modification amount is $\delta_{A}=0.005\;mm$; *B* point modification amount is $\delta_{B}=0.003\;mm$; *P* point modification amount is $\delta_{P}=0\;mm$; *C* point modification amount is $\delta_{C}=0.003\;mm$; *D* point modification amount is $\delta_{D}=0.005\;mm$; contact trace modification coefficient is $a_{v0}=0\;mm$; contact trace modification coefficient is $a_{v1}=0\;mm$; contact trace modification coefficient is $a_{v2}=1.9875\times 10^{-4}\;mm$; contact trace modification coefficient is $a_{v3}=0\;mm$; and contact trace modification coefficient is $a_{v4}=1.9634\times 10^{-4}\;mm$.

It can be seen from Figure 8a,b that, under the condition of case 1 invariable, the strength of the pinion gear along the tooth width direction is significantly lower than that of the face gear. With the increase of pressure angle and displacement coefficient, the difference in bending strength between the pinion gear and face gear along the tooth width direction gradually decreases, as shown in Figure 8c–j. It can be seen from Figure 8k,l that under the conditions of case 6, the difference in bending strength between the pinion gear and the face gear is greatly reduced. Since the pinion gear is a standard cylindrical gear and is easy to replace, the strength of the pinion gear can be appropriately lower than that of the face gear. As a result, case 6 is finally determined as the final design parameter of the face gear pair.

## 4. Electrochemical Machining and Transmission Performance Test

### 4.1. Electrochemical Machining Test of Profile Shifted Face Gear

The face gear material is 18CrNi4A, and the chemical composition of the material measured by the Hopkinson pressure bar experiment is shown in Table 2.

**Figure 9:**
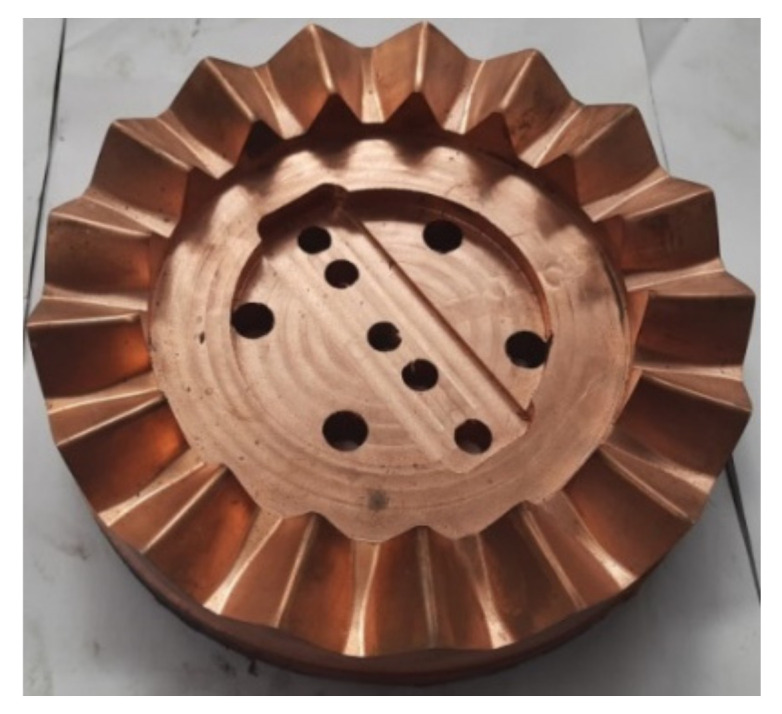
Electrochemical machining cathode for face gear.

High-frequency quenching is performed on the sample of the face gear after milling. To ensure the quality of the face gear tooth surface after electrochemical machining, the tooth surface machining allowance is set to 0.3 mm. To save processing costs and improve processing efficiency, electrochemical machining is divided into two steps: rough machining and finishing machining. Rough machining graphite electrodes and finishing machining copper electrodes are developed respectively. Graphite electrodes are cheaper and faster in processing loss, and can be used for electrochemical rough machining. The copper electrodes are relatively expensive and stable during processing, and can be used in fine machining. The rough machining allowance of the graphite electrode is 0.2 mm, and the finishing machining allowance of the copper electrode is 0.1 mm. The electrochemical finishing electrode of the face gear is shown in Figure 9.

**Figure 10:**
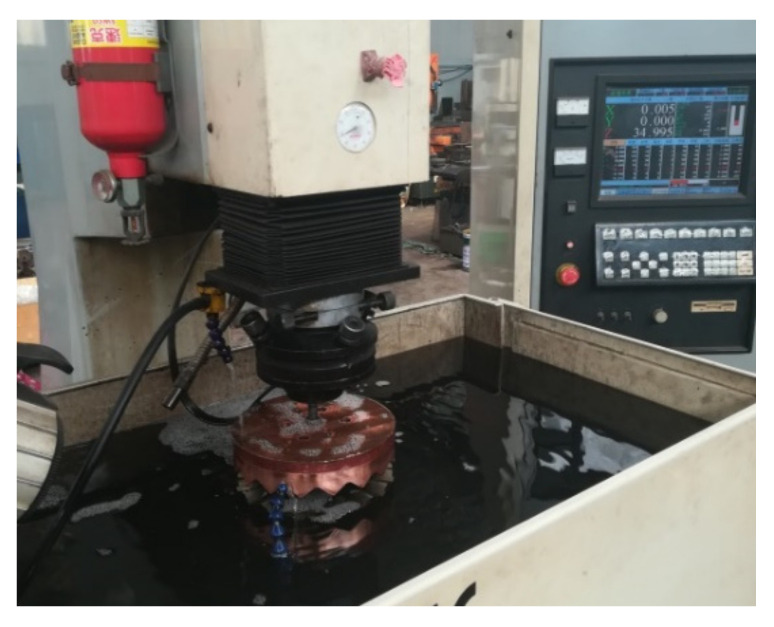
Electrochemical machining process of face gear.

**Figure 11:**
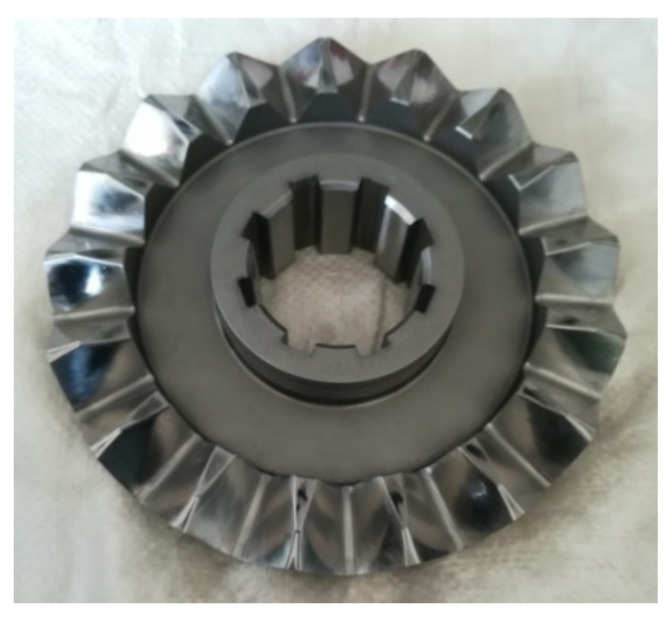
Face gear after processing.

The selected equipment for electrochemical machining of face gear is Taiwan Meixi Spark Machine, model 430. According to the structural characteristics of the machine tool and the face gear workpiece, the cathode tool and the face gear workpiece positioning fixtures are developed for precise clamping and positioning of the cathode and face gear in electrochemical machining. After the positioning of the face gear and the electrochemical cathode is completed, the electrochemical precision machining test of the face gear is carried out according to the electrochemical machining parameters of the face gear, as shown in Table 3. The electrochemical machining process is shown in Figure 10, and the face gear after electrochemical machining is shown in Figure 11.

**Figure 12:**
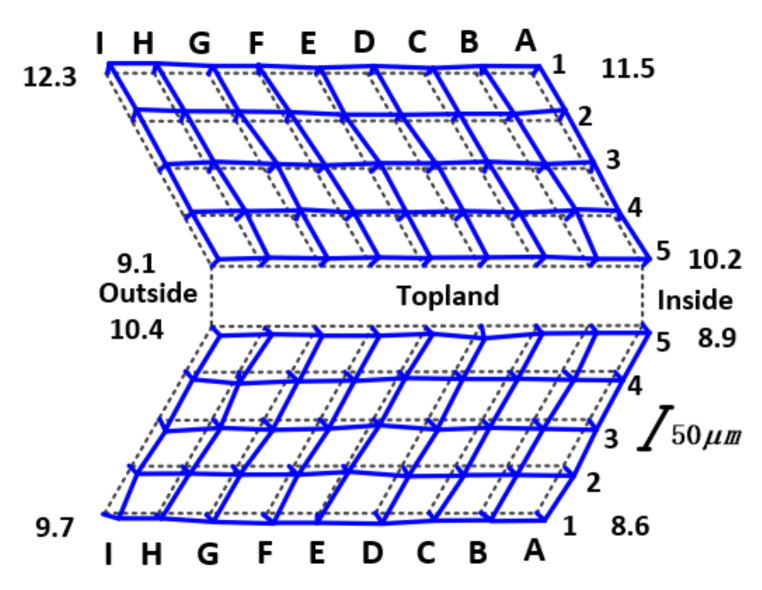
Flank topology of the face gear after finishing.

The topological diagrams of the face gear tooth surface after electrochemical machining are shown in Figure 12. It can be seen from the figure that the tooth surface deviation is small, and the tooth surface accuracy is higher than that after grinding [19].

### 4.2. Electrochemical Machining Test of Profile Shifted Face Gear

To verify the performance of the face gear transmission, the reliability test verification is carried out to simulate the actual working conditions of the face gear transmission system. The test time of one cycle is 20 h, a total of ten cycles are carried out, and the cumulative time of the test is 200 h. A cyclic process of the transmission box, simulating the speed and torque changes of each gear under actual working conditions, and detecting the efficiency and temperature changes during the transmission process. The test box is a gearbox with a reduction ratio of 17/12. The maximum output speed of the motor is 1500 r/min. To increase the speed, a speed-increasing box is connected after the motor is output.

**Figure 13:**
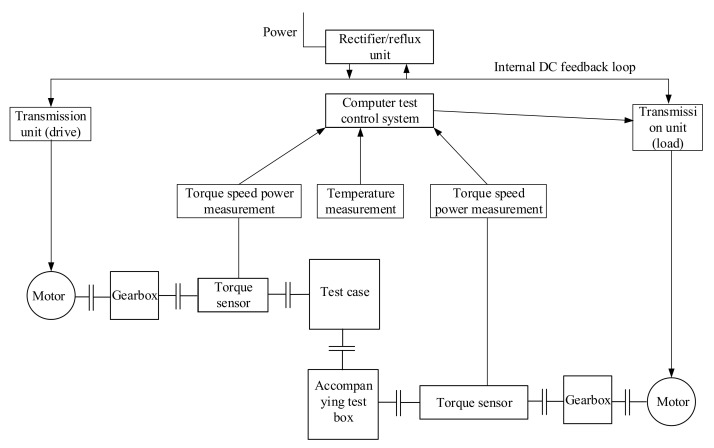
Schematic diagram of test bench layout.

**Figure 14:**
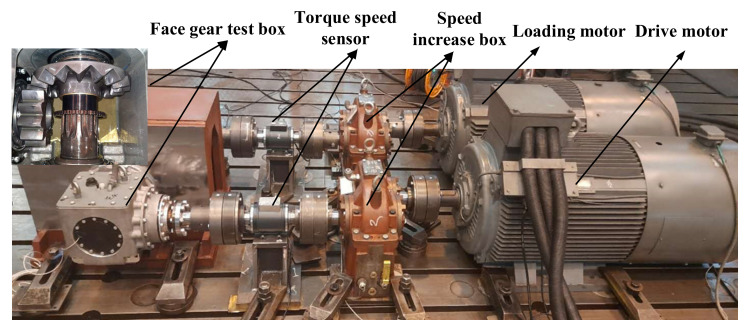
Gearbox test bench.

The layout of the test bench mainly includes: drive motor, speed-increasing box, loading motor, torque sensor, computer test control system, test box, accompanying test box, and coupling, etc. The layout diagram of the test bench is shown in Figure 13, and the reliability test bench of the face gear is shown in Figure 14.

The test bench adopts the DC side feedback AC variable frequency drive mode, analyzes the mathematical model of the AC motor under the stator coordinates, and controls the flux linkage and torque of the motor. The space vector is used to analyze the mathematical model of the three-phase AC motor and control various physical quantities. The power supply of the system is the secondary power supply of the power supply transformer: the capacity is 100 KVA, the voltage is 380 V, and the 540 VDC power is output after switching and rectification. The DC power is inverted by the frequency conversion cabinet and drives the drag motor to work, and the drag motor drives the tested gearbox to run. The tested gearbox and the accompanying test gearbox adopt a back-to-back connection. The tested gearbox drives the accompanying test gearbox to run, and the accompanying test gearbox drives the load motor to run. The load motor works under power generation conditions, and the generated electrical energy is fed back by the load inverter. On the DC 540 VDC side, the electric energy is driven by the drive inverter to drive the drive motor. During the test, the load of the face gear transmission system can be realized by controlling the speed of the driving motor and the torque of the load motor.

**Figure 15:**
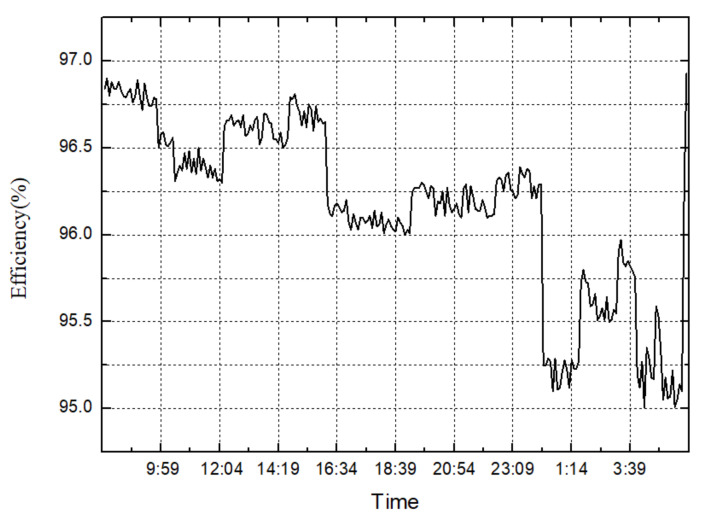
Temperature change curve in one cycle.

**Figure 16:**
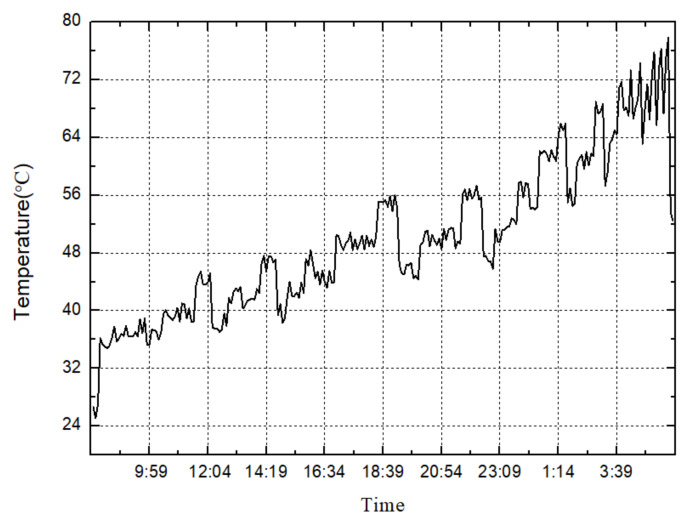
Efficiency change curve in one cycle.

The transmission efficiency and temperature change curves, after the reliability test, are shown in Figure 15 and Figure 16. It can be seen from Figure 15 that, as the gear increases, the speed gradually increases, the torque gradually decreases, and the overall transmission efficiency shows a downward trend. The efficiency jump at the end of the cycle is caused by the climbing condition, where input speed is 160 r/min and input torque is 2910 N.m. It can be seen from Figure 16 that, as the gear increases, the speed gradually increases, and the torque gradually decreases. The gearbox temperature shows an overall upward trend. The plunge in temperature at the end of the cycle is also caused by the climbing condition. During the entire 200-h test process, the face gear transmission has stable transmission performance and stable operation. After the reliability test of the transmission box, shown in Figure 14, the appearance of the gear pair is intact and there is no bad condition after opening the box. Compared with the previous transmission test standards and data, the results of the face gear reliability test meet the service performance requirements of heavy vehicles.

## 5. Conclusions

To solve the problems of face gear pair strength performance considering service space limitations, this paper proposed the strength matching design method of the profile-shifted face gear pair.

(1) According to the meshing principle of face gear, a contact trace reverse modification method was proposed to improve the strength performance of the face gear pair.
(2) By analyzing the strength performance of the face gear pair under different parameters, the optimization parameters of the face gear pair based on strength matching were obtained.
(3) According to the characteristics of materials, the machining of face gear by electrochemical machining was carried out, and the precision and consistency of the tooth surface were better than that of grinding.
(4) The experimental results of face gear pair transmission showed that the transmission performance was stable and the strength performance was effectively improved after strength matching, and there were no abnormal phenomenon, such as tooth surface wear, before.
(5) The method presented in this paper could be applied to the engineering popularization of other new transmission systems.

## Figures and Tables

**Table 1:** Design parameters of profile shifted face gear.

Parameters	Case 1	Case 2	Case 3	Case 4	Case 5	Case 6
Module	9	9	9	9	9	9
Pressure angle	25°	25°	25°	30°	30°	30°
Tooth number of the face gear	17	17	17	17	17	17
Tooth number of the pinion	12	12	12	12	12	12
Modification coefficient	0	0.1	0.2	0.3	0.4	0.5
Tooth width	20 mm	20 mm	20 mm	30 mm	30 mm	30 mm
Equivalent input torque	3000 N∙m					
Rotation rate	183 r/min					

**Table 2:** Chemical composition of 18CrNi4A.

C	Mn	Si	S	P	Cr	Ni
0.15~0.20	0.30~0.60	≤0.35	≤0.010	≤0.015	0.80~1.10	3.75~4.25

**Table 3:** Electrochemical machining parameters of face gear.

Parameters	Value
Electrochemical volume equivalent	2.22 mm^3^/(A ∙ min)
Processing temperature	(30 ± 2) °C
Electrolyte formula	25% NaNO_3_ + 75% NaCl
Electrolyte concentration	7.5%
Conductivity of electrolyte	0.0095 (Ω ∙ mm)^−1^
Processing voltage	8 V
Feed rate	0.5 mm/min
Current efficiency of electrolyte	70%
Feed depth	1 mm

## Data Availability

Not applicable.

## Abbreviations

α	Pressure angle
u	Variable parameter
$r_{1}$	Rack tooth surface equation
$r_{2}$	Displacement gear tooth surface equation
$M_{21}$	Coordinate transformation matrix of $S_{1}$ to $S_{2}$
$f\left(u,\varphi \right)$	Meshing equation
$v_{1}^{\left(12\right)}$	Relative speed
$r_{bs}$	Base circle radius of the shaper
$u_{s}$	Tooth surface parameter marked in the $z_{s}$ direction
$\theta_{s}$	Involute parameter
$\theta_{os}$	Parameter used to determine the width of the tooth groove of the shaper on the base circle
$r_{s}$	Reference radius of the shaper
$N_{s}$	Number of shaper teeth
$v^{\left(s2\right)}$	Relative speed
$M_{2s}$	Conversion matrix
μ	Angle between the reverse contact trace $L_{m}$ and the projected coordinate axis $X_{g}$
ξ	Angle between the tooth contact line $L_{c}$ and the projected coordinate axis $X_{g}$
${l^{\prime }}_{g}$	Amount of variation
$\delta_{h}$	Amount of modification along the contact line of the tooth surface
